# Expression of the inhibitory receptor NKG2A correlates with increased liver and splenic NK cell response to activating receptor engagement

**DOI:** 10.1002/iid3.156

**Published:** 2017-03-24

**Authors:** Claire E. Meyer, Phillip N. Key, Toby Zhu, Mark Shabsovich, Ann Ni, Sandeep K. Tripathy

**Affiliations:** ^1^ Gastroenterology DivisionDepartment of MedicineWashington University School of MedicineSt. LouisMissouriUSA

**Keywords:** Tolerance, microenvironment, cytokines, adaptation, leukocytes

## Abstract

**Introduction:**

Natural killer (NK) cells play a critical role in the innate immune response to viruses and tumors, and comprise a large proportion of the hepatic lymphocyte population. They must remain tolerant to non‐pathogenic antigens while protecting the host from harmful agents. Herein, we investigate how the NK cell response to activation receptor engagement is altered in the liver.

**Methods:**

In this study, we assess IFN‐γ production and degranulation of splenic NK cells and selected subsets of liver NK cells. Flow cytometry (FCM) was used to asses IFN‐γ production and degranulation following stimulation of the NK cells with plate bound antibodies to activating receptors.

**Results:**

We show that smaller percentages of hepatic NK cells produce interferon (IFN)–γ and/or degranulate than do splenic NK cells upon stimulation through activating receptors. We also found that smaller percentages of the circulating NK (cNK) cells in the liver produce IFN‐γ and/or degranulate, compared to the liver tissue resident NK (trNK) cells. In addition, IFN‐γ production by liver cNK cells is not increased in IL‐10 deficient mice, suggesting that their hyporesponsiveness is not mediated by the presence of this anti‐inflammatory cytokine in the hepatic microenvironment. On the other hand, liver trNK cells express higher levels of the inhibitory receptor NKG2A than do cNK cells, correlating with their increased IFN‐γ production and degranulation.

**Conclusions:**

Liver cNK cells’ hyporesponsiveness to stimulation through activating receptors is independent of IL‐10, but correlates with decreased NKG2A expression compared to trNK cells. In addition, we demonstrate that liver NK cells become further hyporesponsive upon continuous engagement of an activating receptor on their cell surface.

## Introduction

Natural killer (NK) cells play an important role in the innate immune response to a number of pathogens and are among the first cells to generate an immune response. They function by destroying abnormal cells through the secretion of cytotoxic granules, as well as secreting cytokines which shape the subsequent immune response 1. While most often studied in the spleen, NK cells are found in other solid organs including the thymus, uterus, and liver 2, 3, 4, 5, 6. NK cells in these solid organs can be differentiated from each other by function, cell surface markers, and transcription factors required for development 2.

Based on the mutually exclusive expression of two cell surface markers, NK cells within the liver can be divided into two populations. One population, tissue resident (tr) NK cells, express CD49a but not CD49b (both are integrin alpha subunits). The other population, conventional or circulating (c) NK cells, transit through the liver and express CD49b but not CD49a 2. The trNK cells also differ from cNK cells in that they do not express several Ly49 receptors at as high a level. In addition, the level of NKG2A expression is higher on trNK cells as compared to cNK cells. However, it appears that trNK cells as well as cNK cells produce similar levels of IFN‐γ upon stimulation with phorbol 12‐myristate 13‐acetate (PMA) and ionomycin 2, 3, 7.

Previous studies have suggested that liver NK cells are hyporesponsive compared to splenic NK cells based on their dampened IFN‐γ response to IL‐12/IL‐18 stimulation 8, 9. Their work demonstrated that within the liver there is a population of NK cells that lack expression of Ly49H and express higher levels of the inhibitory receptor NKG2A. It also suggested that the increased expression of NKG2A resulted in decreased function of the NK cells. While this study did not differentiate between the circulating versus trNK cells 2, the Ly49H^−^, NKG2A^+^ population of NK cells described likely represents the trNK cells of the liver that have been described more recently 2, 3, 4, 10.

In contrast to these prior studies, which assessed activation of NK cells using cytokines, in the present study, we sought to identify the factors involved in determining the response of liver NK cells to engagement of their activating receptors. We showed that smaller percentages of NK cells isolated from the liver produce IFN‐γ and/or degranulate when stimulated through activating receptors than do splenic NK cells, and that this functional alteration within the liver is limited to the cNK cells. In addition, IFN‐γ production by liver cNK upon stimulation cells is not increased in IL‐10 deficient mice, suggesting that IL‐10 does not mediate NK cell hyporesponsiveness within the hepatic microenvironment, as has been previously proposed. We found that increased IFN‐γ production and degranulation by trNK cells, compared to cNK cells, correlates with increased expression of the inhibitory receptor NKG2A. Finally, using an m157‐transgenic mouse model system, we demonstrated that continuous engagement of the Ly49H activating receptor results in a further dampened IFN‐γ response of Ly49H^+^ liver NK cells to engagement of the NK1.1 activation receptor.

## Results

### Smaller percentages of liver NK cells produce IFN‐γ and/or degranulate compared to splenic NK cells as a result of defects seen in the circulating liver NK cell population

**Figure 1:**
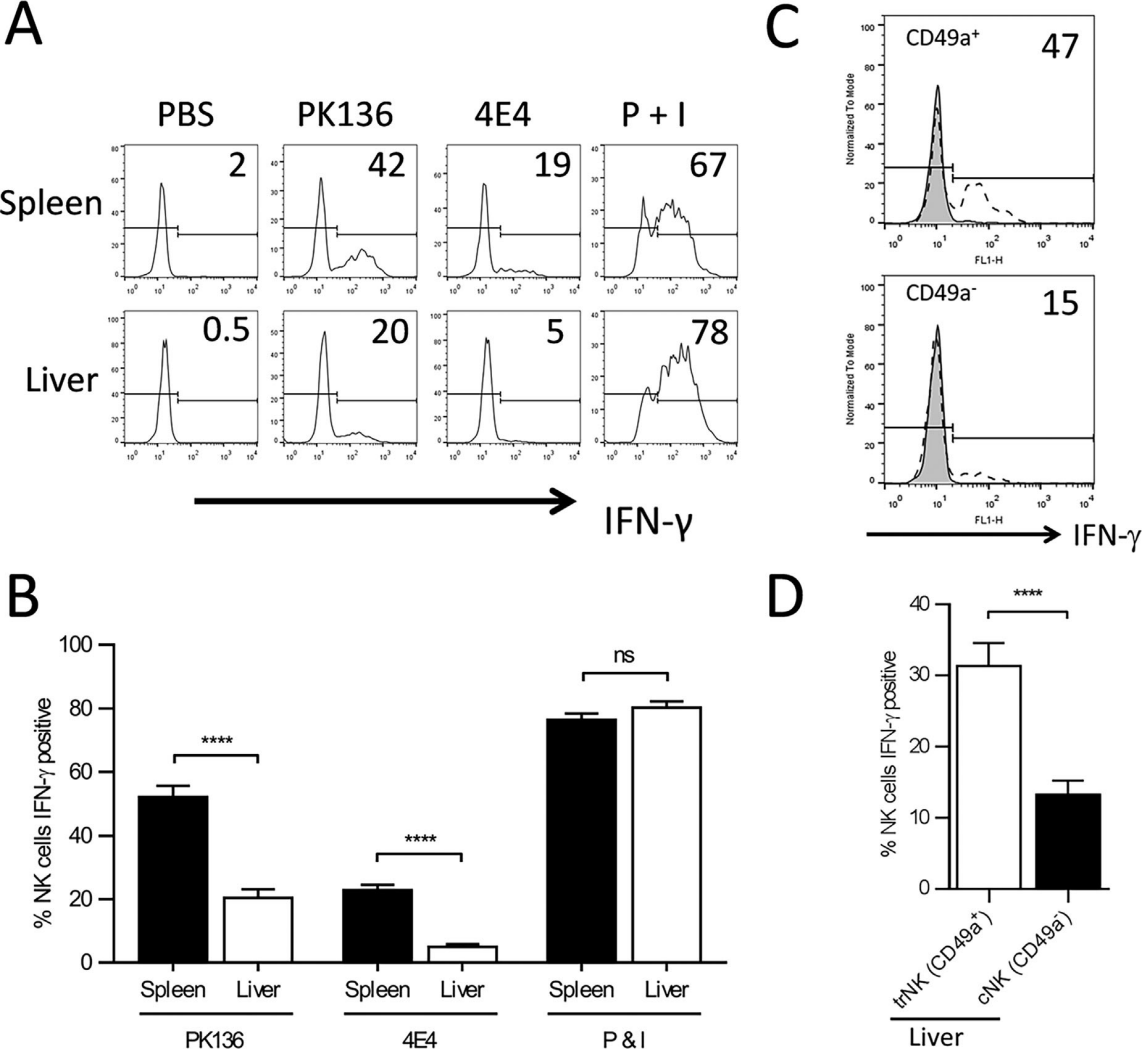
Liver NK cells produce less IFN‐γ than splenic NK cells as a result of defects seen in the circulating liver NK cell population. (A) Representative histogram demonstrating IFN‐γ production by freshly isolated lymphocytes from the spleen and liver of WT mice stimulated with PBS, plate bound PK136 (αNK1.1), plate bound 4E4 (αLy49D), or PMA and ionomycin (P + I). The plots were gated on NK cells (NK1.1^+^, CD3^−^). (B) The combined average percentage of IFN‐γ producing NK cells from the spleen (black column, *n* = 6) and liver (white column, *n* = 6) of WT mice stimulated with plate bound PK136, 4E4, or P + I. The results are presented as the mean ± SEM (****p < 0.0001; ns, not significant). (C) Representative histograms demonstrating IFN‐γ production by freshly isolated liver lymphoctyes from a WT mouse stimulated with plate bound PK136 (αNK1.1). The histograms are gated on NK cells (NK1.1^+^, CD3^−^), and the numbers represent the percentage of IFN‐γ producing CD49a^+^ or CD49a^−^ NK cells. The tinted histograms are PBS‐stimulated negative controls and the open dashed histograms represent PK136‐stimulated NK cells. (D) The combined average percentage of IFN‐γ producing CD49a^+^ NK cells and CD49a^‐^ NK cells from WT mice (*n* = 8) stimulated with plate bound PK136. The results are presented as the mean ± SEM (****p < 0.0001; ns, not significant).

NK cells were harvested from the liver and spleen of wild‐type (WT) mice and assessed for the production of IFN‐γ following stimulation with plate‐bound antibodies to individual activating receptors (NK1.1 or Ly49D). When stimulated through NK1.1, a significantly lower percentage of liver NK cells produced IFN‐γ, compared to splenic NK cells (Fig. 1A and B). This was not due to a difference in the expression of the activating receptor NK1.1 as the mean fluorescence intensity (MFI) of NK1.1 expression on liver NK cells was not significantly different from its expression on splenic NK cells (data not shown). Similarly, when stimulated through Ly49D, a significantly lower percentage of liver NK cells produced IFN‐γ, compared to splenic NK cells (Fig. 1A and B). This suggests that compared to splenic NK cells, liver NK cells produce less IFN‐γ in response to engagement of their activating receptors. Stimulation of liver and splenic NK cells with PMA and ionomycin (P + I) resulted in the production of similar levels of IFN‐γ, suggesting that the defects are specific to stimulation through cross‐linking of the activating receptors.

As the hepatic NK cell population is composed of both trNK cells (CD49a^+^) and cNK cells (CD49a^−^), we sought to determine if both or only one of these populations are hyporesponsive. To evaluate this, liver NK cells from WT mice were stimulated through NK1.1 and assessed for IFN‐γ production by the trNK cell and cNK cell populations. We found when stimulated through NK1.1, a significantly higher percentage of trNK cells produced IFN‐γ, compared to cNK cells (Fig. 1C and D). Similar to previous studies, when the liver NK cells were stimulated with P + I, both populations were able to produce IFN‐γ (data not shown), suggesting that the NK cells can still make IFN‐γ but activation through the engagement of the activation receptor was disrupted. Thus, only liver cNK cells appear to be hyporesponsive to stimulation through activation receptors.

**Figure 2:**
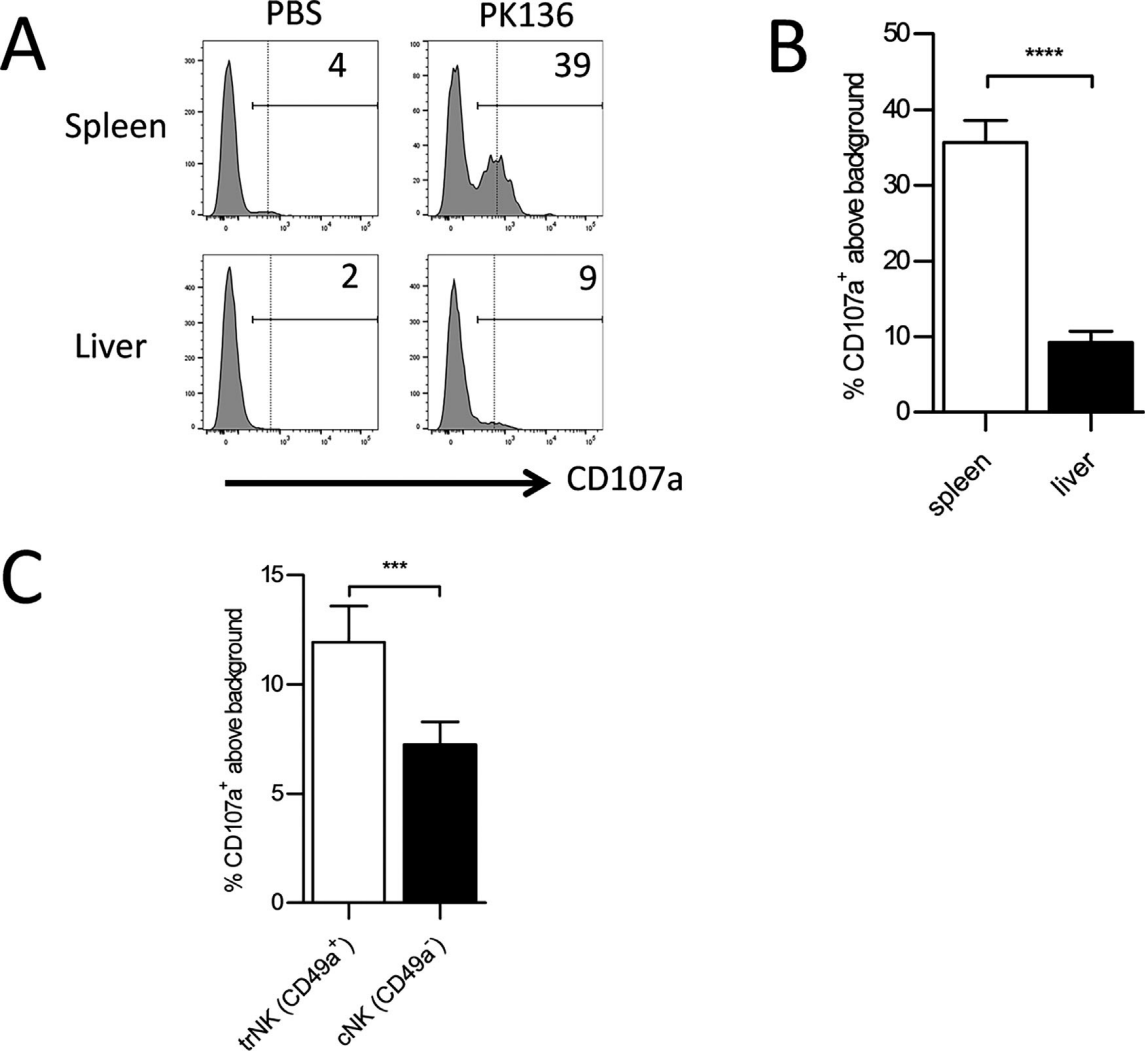
Liver NK cells degranulate less than splenic NK cells as a result of defects seen in the circulating liver NK cell population. (A) Representative histogram demonstrating CD107a expression by freshly isolated lymphocytes from the spleen and liver of WT mice stimulated with PBS or plate bound PK136 (αNK1.1). The plots were gated on NK cells (NK1.1^+^, CD3^−^). (B) The combined average percentage of CD107a^+^ NK cells from the spleen (white column, *n* = 10) and liver (black column, *n* = 10) of WT mice stimulated with plate bound PK136. To calculate %CD107a^+^ above background, the basal percentage of cells with CD107a surface expression in the PBS stimulation was subtracted from the samples stimulated with αNK1.1. The results are presented as the mean ± SEM (****p < 0.0001). (C) The combined average percent CD107a^+^ above background in CD49a^+^,CD49b^−^ NK cells versus CD49a^‐^,CD49b^+^ NK cells from WT mice (*n* = 10) stimulated with plate bound PK136. The results are presented as the mean ± SEM (***p < 0.001). Statistical analyses were performed using paired *t*‐test.

In addition to IFN‐γ production, we also compared degranulation (assessed by expression of CD107a on the cell surface) of NK cells from the liver and spleen in response to plate bound stimulation through NK1.1. We found that a significantly higher percentage of NK cells in the spleen underwent degranulation in response to this stimulation compared to liver NK cells (Fig. 2A and B). In addition, we were able to show that a significantly higher percentage of trNK cells underwent degranulation compared to cNK cells within the liver (Fig. 2C). This was similar to the results seen with IFN‐γ production by the respective NK cells.

### Circulating NK cells from the livers of IL‐10R2ko mice, but not IL‐10ko, mice produce more IFN‐γ when stimulated through NK1.1, compared to those from wild‐type mice

**Figure 3:**
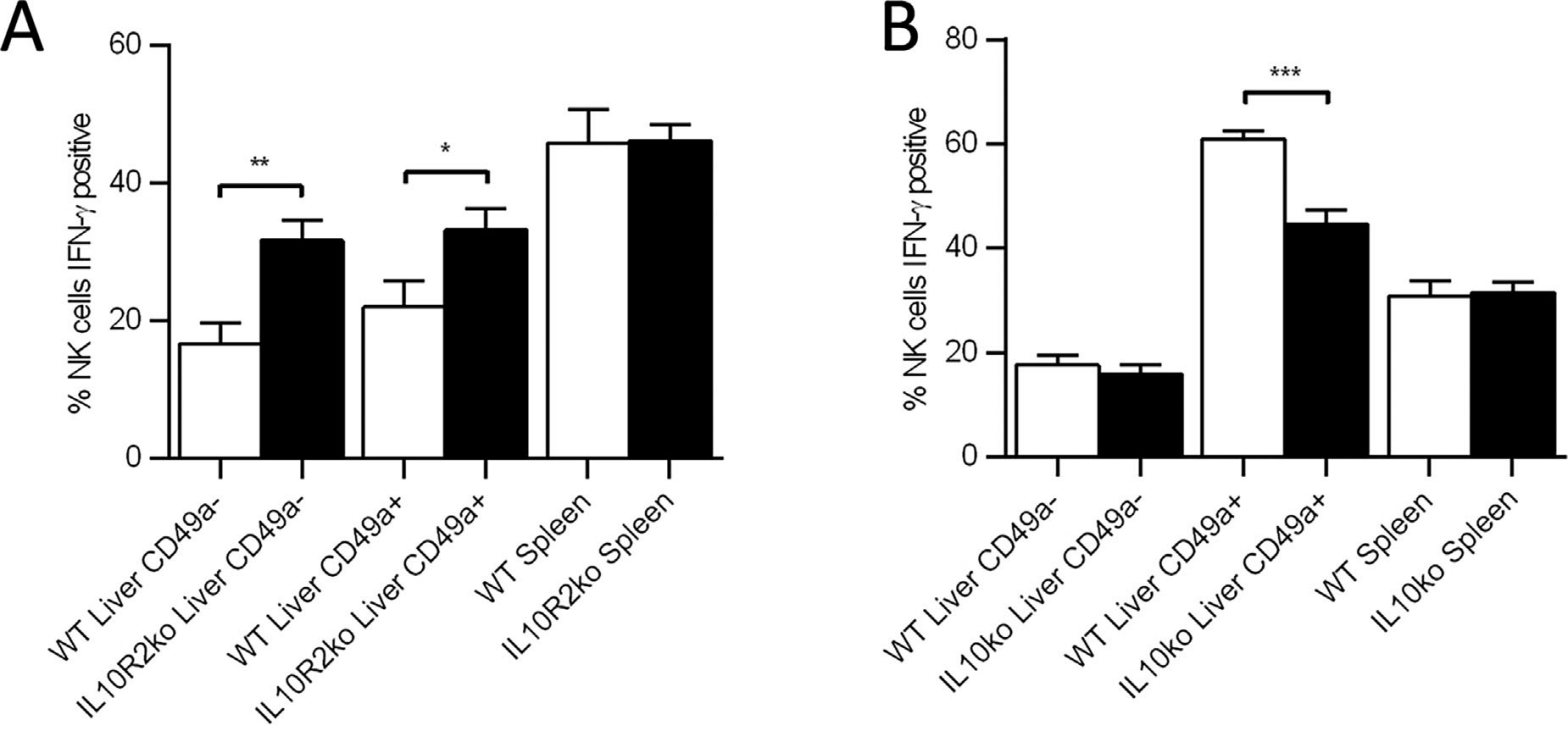
Circulating NK cells from IL‐10R2 ko mice but not IL‐10 ko mice produce more IFN‐γ upon activation receptor stimulation than those from WT mice. (A) The percentage of circulating hepatic NK cells (NK1.1^+^, CD3^−^, CD49a^−^), tissue resident hepatic NK cells (NK1.1^+^, CD3^−^, CD49a^+^), and splenic NK cells (NK1.1^+^, CD3^−^, CD49a^−^) from WT (*n* = 4) and IL‐10R2 ko (*n* = 7) mice that are producing IFN‐γ following stimulation with plate bound PK136 (αNK1.1). (B) The percentage of circulating hepatic NK cells, tissue resident hepatic NK cells, and splenic NK cells from WT (*n* = 8) and IL‐10 ko (*n* = 8) mice that produce IFN‐γ following stimulation with plate bound PK136 (αNK1.1). (*p < 0.05; **p < 0.01; ***p < 0.001).

We assessed the role that the anti‐inflammatory cytokine IL‐10, found in the liver microenvironment, might play in cNK cell function. Hepatic NK cells were harvested from IL‐10R2ko mice (in which the beta subunit of the IL‐10 receptor is not expressed), stimulated through NK1.1, and assessed for IFN‐γ production. IFN‐γ was produced by a significantly higher percentage of liver cNK as well as trNK cells from IL‐10R2ko mice compared to wild‐type mice (Fig. 3A). Splenic NK cells, however, were similar in both groups of mice. This suggests that cytokines utilizing the IL‐10R2 subunit may be involved in the induction of hyporesponsiveness in cNK cells in the liver but are not involved in the spleen.

In addition to the IL‐10 receptor, the IL‐10R2 subunit is shared by the receptors for multiple interleukins, including IL‐22, IL‐26, IL‐28, and IL‐29 11, 12, 13. To look more specifically at the role that IL‐10 might be playing in the induction of hyporesponsiveness, we assessed the production of IFN‐γ by cNK from the liver of IL‐10ko mice. Surprisingly, we did not find a significant difference in the production of IFN‐γ by hepatic cNK cells from IL‐10ko mice compared to those from WT mice (Fig. 3B). Furthermore, we did not observe a difference in IFN‐γ production by splenic NK cells from IL‐10ko mice compared to those from WT mice (Fig. 3B). This suggests that IL‐10 does not play a significant role in determining liver cNK cell function following activation receptor engagement. Of note, we observed decreased IFN‐γ production by trNK cells from IL‐10ko mice as compared to WT (Fig. 3B), suggesting that IL‐10 may alter liver trNK cell function. However, it should be noted that systemically knocked‐out IL‐10 and IL‐10R2 mice were use in these experiments, thus we cannot distinguish if the effects observed above are NK cell intrinsic or due to the loss of expression in another cell type.

### Expression of higher levels of NKG2A corresponds to increased IFN‐γ production and degranulation upon stimulation through the engagement of activating receptors

It has previously been demonstrated that inhibitory receptors can “license” or “educate” NK cells 14, 15, 16, 17, 18. The licensing status of a particular NK cell subset can be determined by assessing intracellular interferon‐gamma production following stimulation with plate‐bound anti‐NK1.1 monoclonal antibody in the presence of brefeldin A 19. Our initial observations demonstrated that a higher percentage of trNK cells produced IFN‐γ upon stimulation through NK 1.1 when compared to cNK cells within the liver. We observed higher levels of NKG2A expression on trNK cells compared to cNK cells (Supplemental Fig. S2), consistent with observations by other groups 2, 20.

**Figure 4:**
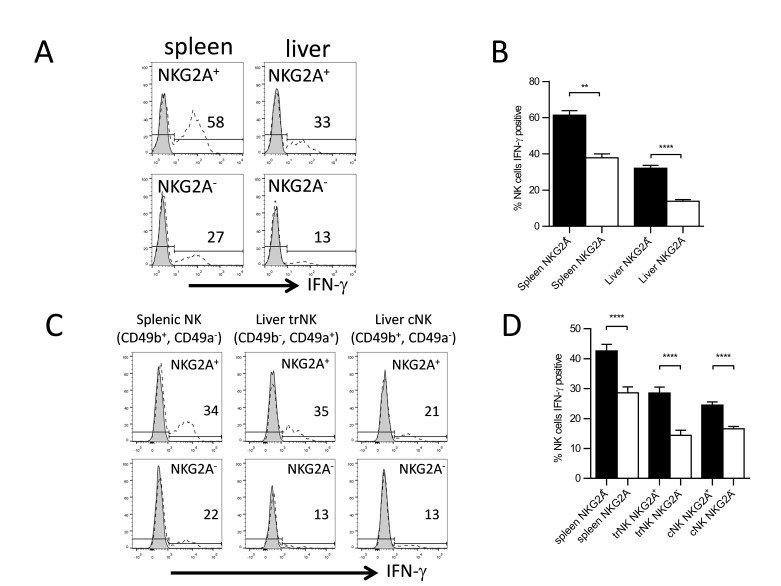
Increased NKG2A expression correlates with increased IFN‐γ production. (A) Representative histograms demonstrating IFN‐γ production by freshly isolated lymphocytes from the spleen and liver of WT mice stimulated with plate bound PK136 (αNK1.1). The histograms were gated on NK cells (NK1.1^+^, CD3^−^). The numbers represent the percentage of IFN‐γ producing NKG2A^+^ or NKG2A^−^ NK cells. The tinted histograms are PBS‐stimulated negative controls and the open dashed histograms represent PK136‐stimulated NK cells. (B) The combined average percentage of IFN‐γ producing NKG2A^+^ and NKG2A^−^ NK cells from the liver and spleen of WT mice (*n* = 7) stimulated with plate bound PK136. (C) Representative histograms demonstrating IFN‐γ production by freshly isolated lymphocytes from the spleen and liver of WT mice stimulated with plate bound PK136. The histograms were gated on splenic NK cells (NK1.1^+^, CD3^−^, CD49b^+^, CD49a^−^), liver trNK cells (NK1.1^+^, CD3^−^, DX5^−^, CD49a^+^), or liver cNK cells (NK1.1^+^, CD3^−^, CD49b^+^, CD49a^−^). The numbers represent the percentage of IFN‐γ producing NKG2A^+^ or NKG2A^−^ NK cells. The tinted histograms are PBS‐stimulated negative controls and the open dashed histogram represent PK136‐stimulated NK cells. (D) The combined average percentage of IFN‐γ producing NKG2A^+^ (black columns) and NKG2A^−^ NK cells (white columns) of the different spleen (*n* = 6) and liver populations (*n* = 9) of NK cells from WT mice stimulated with plate bound PK136.The results are presented as the mean ± SEM (**p < 0.01; ****p < 0.0001).

To address the role that NKG2A plays in educating NK cells we assessed IFN‐γ production of NKG2A^+^ and NKG2A^−^ NK cells in both the spleen and liver following stimulation through the NK1.1 receptor. We observed that in both the spleen and the liver, NKG2A^+^ NK cells produced significantly more IFN‐γ compared to NKG2A^−^ NK cells (Fig. 4A and B). Furthermore, when we subdivided the liver NK cells in to trNK cells (CD49b^−^, CD49a^+^) and cNK cells (CD49b^+^, CD49a^−^), we still observed that a significantly higher percentage of NKG2A^+^ NK cells produced IFN‐γ as compared to NKG2A^−^ NK cells (Fig. 4C and D). As noted earlier, trNK cells expressed lower levels of some Ly49 receptors, including the inhibitory receptor Ly49I. This could partially explain the decreased IFN‐γ production by the cNK cells as compared to the trNK cell population in the liver. Taken together, this suggests, in contrast to previous studies implying that increased NKG2A levels inhibit hepatic NK cell function, that NKG2A engagement might play a role in educating NK cells in both the spleen and the liver 9.

In addition, we observed increased IFN‐γ production by trNK cells from WT mice as compared to IL10ko mice (Fig. 3B). We also observed that trNK cells from WT mice expressed higher levels of NKG2A than trNK cells from IL10ko mice (Supplemental Fig. S1). Thus trNK cells from WT mice produced more IFN‐γ and expressed higher levels of NKG2A than trNK cells from IL10ko mice, further suggesting that expression of NKG2A correlates with increased function of the NK cells.

**Figure 5:**
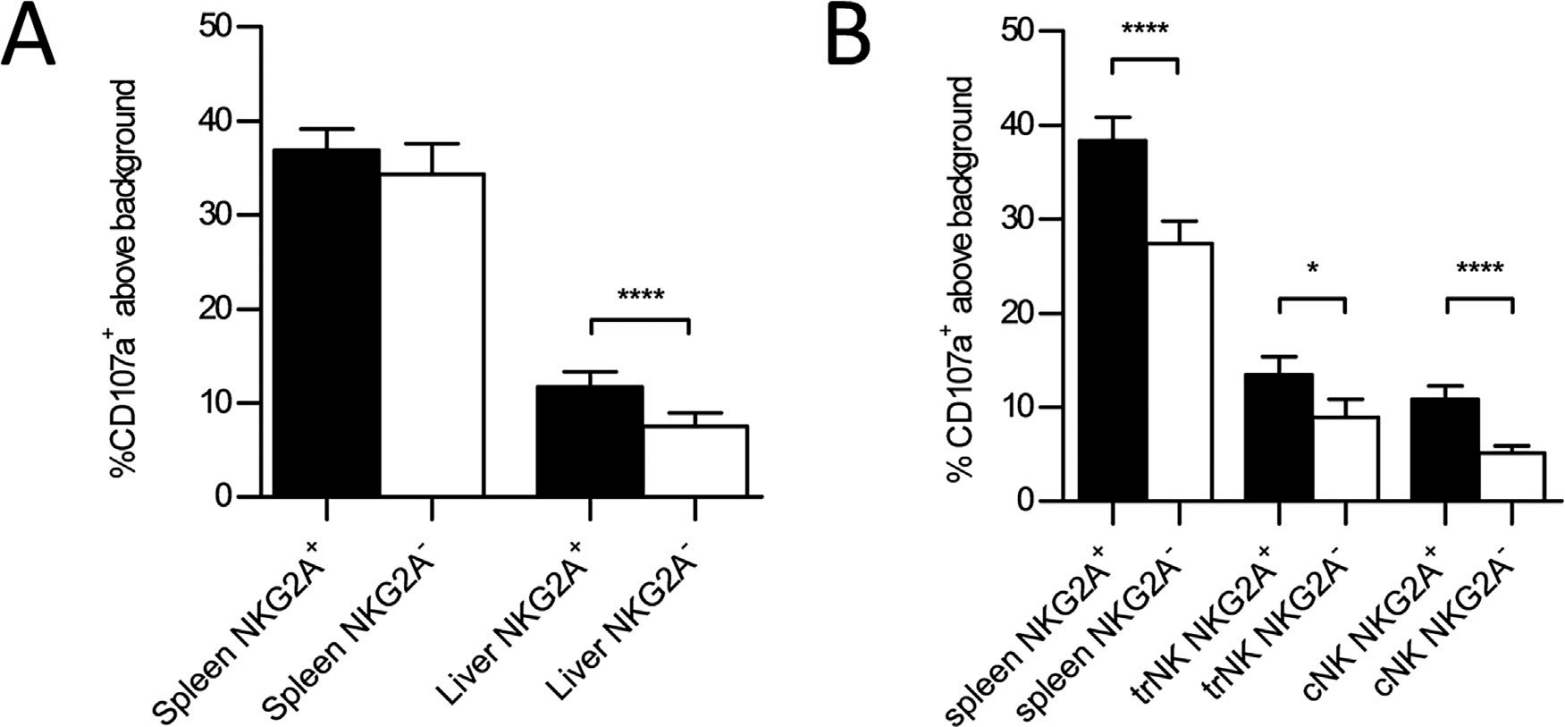
Increased NKG2A expression correlates with increased degranulation. (A) The combined average percentage of CD107a^+^ above background in NKG2A^+^ and NKG2A^−^ NK cells (NK1.1^+^, CD3^−^) from the liver and spleen of WT mice stimulated with plate bound PK136. (B) The combined average percentage of CD107a^+^ above background in NKG2A^+^ (black columns) and NKG2A^−^ NK cells (white columns) of splenic NK cells (NK1.1^+^, CD3^−^, CD49b^+^, CD49a^−^), liver trNK cells (NK1.1^+^, CD3^−^, CD49b^−^, CD49a^+^) or liver cNK cells (NK1.1^+^, CD3^−^, CD49b^+^, CD49a^−^) from WT mice stimulated with plate bound PK136.The results are presented as the mean ± SEM (*p < 0.05; ****p < 0.0001). Statistical analyses were performed using paired *t*‐test on sample size of 10 mice in each group.

Similar to IFN‐γ production, degranulation was also affected by expression of NKG2A on NK cells. In the liver, we saw a significantly higher percentage of degranulation when we compared NKG2A^+^ to NKG2A^−^ NK cells in the total hepatic NK cells population as well as when we broke the hepatic NK cells into their trNK and cNK cell subsets (Fig. 5A and B). The percent degranulation of NKG2A^+^ NK cells was not significantly different in the spleen compared to NKG2A^−^ NK cells when we assessed the NK1.1^+^, CD3^−^ populations (Fig. 5A). However, when we gated on NK1.1^+^, CD49b^+^, CD49b^−^, CD3^−^ cells in the spleen we noted significantly increased degranulation in the NKG2A^+^ NK cells compared to the NKG2A^−^ NK cells (Fig. 5B).

### Continuous engagement of an activating receptor results in decreased IFN‐γ production by liver NK cells

**Figure 6:**
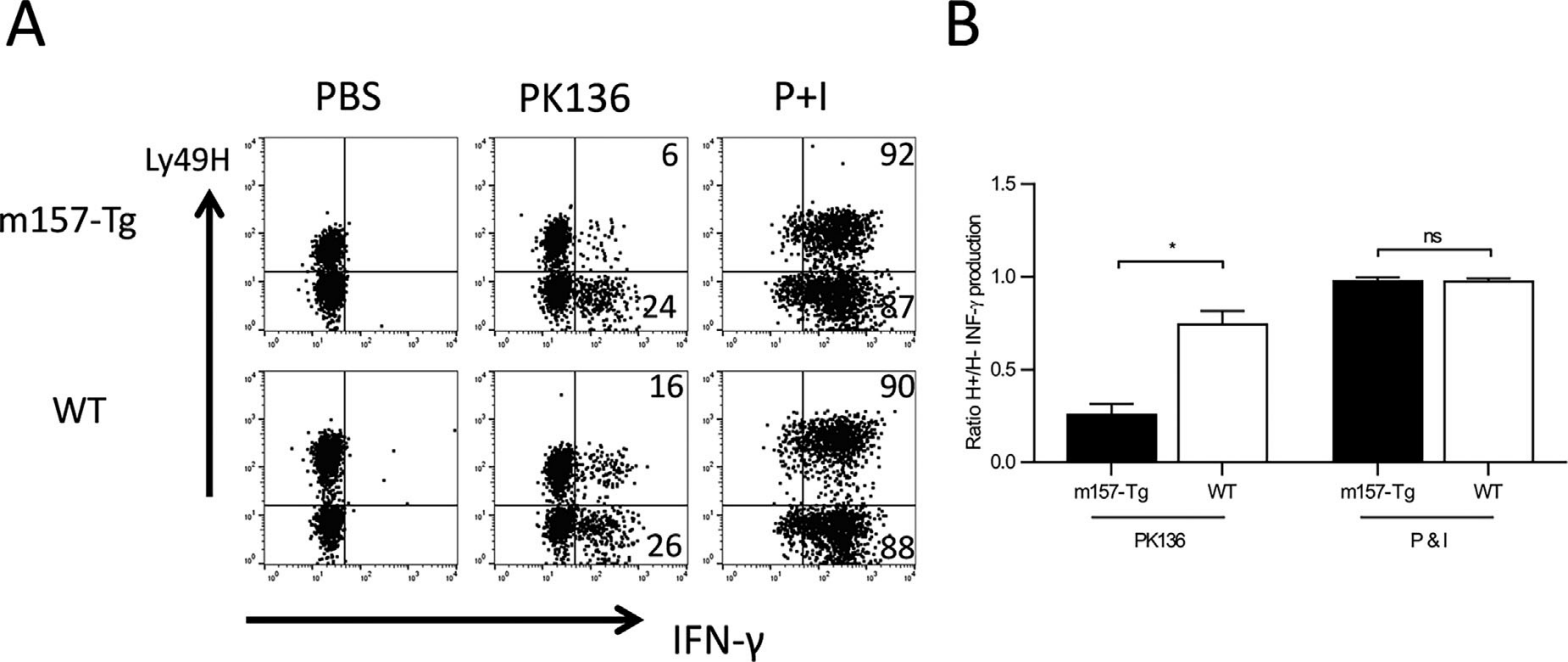
Hepatic NK cells display activation receptor mediated hyporesponsiveness. (A) Representative dot plots demonstrating IFN‐γ production by freshly isolated lymphocytes from the liver of WT and m157‐Tg mice stimulated with PBS (control), plate bound PK136 (αNK1.1) or PMA and ionomycin (P + I). The dot plots were gated on NK cells (NK1.1^+^, CD3^−^), and the numbers represent the percentage of IFN‐γ producing Ly49H^+^ or Ly49H^−^ NK cells. (B) The ratio of the percentage of IFN‐γ producing Ly49H^+^ NK cells to the percentage of IFN‐γ producing Ly49H^−^ NK cells from WT mice (*n* = 8) and m157‐Tg mice (*n* = 10) stimulated with plate bound PK136 or P + I. The results are presented as the mean ± SEM (*p < 0.05; ns, not significant).

Finally, we wanted to determine if continuous engagement of one activating receptor (Ly49H) on NK cells in the liver microenvironment would further decrease the ability of NK cells to be activated via a separate activating receptor (NK1.1). To accomplish this, liver NK cells were harvested from both WT C57Bl6J mice and a transgenic (Tg) overexpressor (m157‐Tg) of the Ly49H receptor's ligand, m157. These were stimulated in vitro with plate‐bound antibody to NK1.1 and assessed for IFN‐γ production. The results are expressed as the ratio of the proportion of Ly49H^+^ NK cells producing IFN‐γ compared to the proportion of Ly49H^−^ NK cells producing IFN‐γ. Because not all NK cells express Ly49H and only the Ly49H^+^ NK cells are altered by the presence of m157, this ratio is expected to be less than one if the continuous engagement of Ly49H results in hyporesponsiveness of the Ly49H^+^ NK cells. In liver NK cells from m157‐transgenic mice, this ratio was significantly lower compared to those from non‐transgenic mice (Fig. 6A and B). When we stimulated the cells with P+I, we observed the ratio of both m157Tg and WT mice to be one, suggesting that H^+^ NK cells from m157‐Tg livers functioned normally upon stimulation with P+I. (Fig. 6A and B). This is similar to previous work assessing function of splenic NK cells from m157‐Tg and WT mice 21, 22. This indicates that, similar to splenic NK cells, upon continuous engagement of the Ly49H activating receptor, liver NK cells become hyporesponsive to stimulation through the NK1.1 activation receptor.

## Discussion

NK cells are an important lymphocyte subset of the innate immune system. In addition to being found in the spleen and circulating in the blood, NK cells can be found in a number of solid organs. In fact, NK cells make up a large proportion of the hepatic lymphocyte population 23, 24, 25. Recent work suggests that NK cells in different organs function differently and may derive from distinct lineages 2, 3, 4, 10. This appears to be the case in the liver, where NK cells can be divided into two populations based on the mutually exclusive expression of two cell surface markers. These distinct populations represent tissue resident NK cells and circulating NK cells.

In addition to cell surface markers, several studies have demonstrated that tissue resident NK cells have different transcription factor requirements than circulating NK cells and likely arise from different progenitor cells 2, 26, 27. Linear tracing studies demonstrated that PLZF^high^ cells were a precursor cells to NK1.1^+^, CD49b^−^ NK cells residing in the liver (trNK cells) but not classical circulating NK cells (cNK cells) 27. Another group identified a Lin^−^, ID2^+^, IL‐7Rα^+^, CD25^−^, α4β7^+^, Flt3^−^ precursor that gave rise to all helper‐like innate lymphoid cells (ILCs, which include trNK cells) but not to cNK cells 26. Finally, it has been demonstrated that cNK cells are Eomes^+^, T‐bet^+^ while trNK cells are Eomes^−^, T‐bet^+^ 2. Taken together, this suggests that trNK cells (group 1 ILCs) are a distinct lineage from cNK cells seen in the liver or spleen.

The liver microenvironment provides a unique situation for the immune system in that it must be able to respond to harmful pathogens while at the same time remain tolerant to the many harmless antigens derived from commensal and food sources from the gut 28, 29. There are a number of mechanisms believed to be involved in tolerance in the hepatic immune system, including the induction of regulatory T cells, regulatory Kupffer and dendritic cells, and the presence of immunomodulatory cytokines 30, 31. Several of these are likely involved in altering hepatic NK cell function.

In this study, we demonstrate that hepatic NK cells produced significantly less IFN‐γ and degranulate less than NK cells from the spleen when stimulated through the engagement of cell surface activating receptors. In addition, NK cells from the hepatic microenvironment become further hyporesponsive (dampened IFN‐γ production upon stimulation of the NK1.1 activating receptor) when the Ly49H activating receptor is continuously engaged. This is similar to the activation‐receptor mediated hyporesponsiveness previously demonstrated in splenic NK cells 21, 22.

Previous studies have shown dampened response of hepatic NK cells to cytokine stimulation or treatment with PMA and ionomycin 2, 32. While one of these studies found that cNK and trNK cells produced similar levels of IFN‐γ in response to PMA and ionomycin 2, another showed that cNK cells produced more IFN‐γ than trNK cell 32 under similar conditions. Yet another study assessed cytokine production by Eomes^−^ (corresponding to trNK cells) and Eomes^+^ (corresponding to cNK cells) NK cells within the liver. Although the Eomes^−^ NK cells within the liver produced a wide variety of cytokines, they appeared to produce less IFN‐γ as compared Eomes^+^ NK cells upon stimulation through activating receptors 33. In contrast to these studies, we demonstrate that hepatic cNK cells are defective compared to trNK at producing IFN‐γ and degranulation when stimulated through an activating receptor (Figs. 4 and 5). This pattern is consistent with prior work which demonstrated that in NK cells isolated from human livers, CD49a^+^ NK cells produced more IFN‐γ compared to CD49a^−^ NK cells upon stimulation with PMA and ionomycin 34. Taken together, this suggests that hepatic trNK cells (ILCs) have an intrinsically different response to activating receptor stimulation than do cNK cells within the liver.

Our studies revealed that the hyporesponsiveness of the total hepatic NK cell population is due to the presence of hyporesponsive cNK (CD49a^−^) cells and not trNK cells (CD49a^+^) NK cells. In fact, our data suggest that a higher percentage of liver trNK cells produce more IFN‐γ and degranulate more when compared to liver cNK cells. In addition, a smaller percentage of cNK cells within the liver produced IFN‐γ and/or degranulate compared to cNK cells from the spleen suggesting that upon entering the liver from the circulation, cNK cells become hyporesponsive.

There did not appear to be a difference in the relative abundance of inhibitory Ly49 receptor family memebers when comparing the cNK cells and splenic NK cells; however, differences were noted between cNK and trNK cells within the liver (Supplemental Fig. S2). Similar to previous work, we found that NKG2A levels are comparable on the splenic NK cells and liver cNK cells 2. There is, however, a higher percentage of NKG2A on the liver trNK cells compared to the liver cNK cells and splenic NK cells. Thus, altered NKG2A expression may explain in part the functional differences between liver trNK and cNK cells, but not the differences between hepatic and splenic cNK cells.

Studies assessing liver NK cells following adenoviral infection suggest that the CD49a expression may be difficult to use as a marker to differentiate cNK cells from trNK cells due to the fact that CD49a is transiently upregulated during activation of NK cells in vivo 20. However in our experiments, we assessed the function of NK cells in vitro following plate bound stimulation of lymphocytes from the spleen or liver. We then assessed IFN‐γ production or degranulation (CD107a expression) on the different subsets of NK cells based on cell surface markers. In our experiments, we did not see a meaningful difference in cell surface expression of CD49a on liver NK cells when comparing NK1.1‐stimulated to PBS controls (Supplemental Fig. S3).

NKG2A is a C‐type lectin‐like inhibitory receptor that interacts with the non‐classical MHC class I molecule Qa‐1b (human HLA‐E) 35, 36. The expression of this ligand is dependent on peptides derived from classical MHC class I molecules; thus its expression correlates with the overall expression of MHC class I. Both IL‐10 and TGF‐β have been shown to induce NKG2A expression 8, 9. Other studies suggest that IL‐12 can induce NKG2A expression on NKG2C^+^ NK cells, possibly providing a negative feedback mechanism 37. It has been suggested that the NKG2A population of NK cells might be maintained by the high level of the immune suppressive cytokine IL‐10 present in the liver. Hyporesponsiveness of these NK cells was demonstrated by a dampened IFN‐γ response to IL12/18 stimulation 8, 9.

The increased function of the liver trNK cells in the setting of elevated levels of the inhibitory receptor NKG2A on their cell surface seems counterintuitive and is in contrast to prior work suggesting that increased expression of NKG2A on liver NK cells is responsible for their hyporesponsiveness 8, 9. Our data, suggesting that liver trNK cells produce more IFN‐γ, might be explained by the phenomena of “licensing” or “education” of the NK cells. This is a process in which NK cells expressing inhibitory receptors for self‐MHC class I molecules become functionally competent to be triggered through their activation receptors. Previous work has demonstrated the expression of inhibitory receptors that contain immunoreceptor tyrosine‐based inhibitory motifs (ITIMs) on the cell surface of the NK cell provides a signal that allows for full functional competence of the NK cell 14, 15. The NKG2A receptor contains two ITIMs in its cytoplasmic domain 35, 38. Thus, the higher levels of NKG2A on the liver trNK cells could better “educate” the trNK cells and result in increased IFN‐γ production upon stimulation through activation receptors.

Several other studies corroborate the idea that NKG2A may in fact play a role in “educating” NK cells. It has been shown that NK cells which lack KIRs and NKG2A expression have decreased function and appear developmentally immature 39. Other work suggests that NKG2A expression may be increased on human NK cells that lack self‐recognizing killer‐cell immunoglobulin‐like receptors (KIRs), providing an inhibitory receptor that could be used to educate the NK cells by interacting with self MHC and acquire function 40, 41. In addition, several studies assessing degranulation of human NK cells in response to the K562 cell line demonstrated that CD94/NKG2A plays a major role in normal NK cell education, both independent of and in coordination with expression of KIRs 42, 43. Thus expression of the “inhibitory” receptor NKG2A can result in increased function of the NK cell.

Another study demonstrated that NK cells freshly isolated from surgical specimens from patients with hepatic malignancies have less cytolytic activity compared to those from autologous blood 44. In this study, they observed a higher proportion of CD16^+^ NK cells within the liver that expressed a decreased number of inhibitory receptors 44. This resulted in the liver containing a decreased number of “licensed” NK cells expressing inhibitory receptors compared to blood and could explain their decreased cytolytic function. Although there are clear differences between this study and ours (NK cells from hepatocellular cancer patient vs. healthy mouse), the fact that the function of a population of NK cells in the liver may be controlled by inhibitory receptor engagement is suggested by both.

IL‐10 has been shown to have protective effects in multiple animal models of liver injury 45, 46, 47. IL‐10 has also been shown to have immunosuppressive properties and inhibit IFN‐γ and TNF production by NK cells in vitro 48. Studies have demonstrated that Kupffer cells (liver macrophages) can secrete inhibitory factors, such as IL‐10, as well 49. It has been suggested that IL‐10 in the hepatic microenvironment results in decreased function of liver NK cells in response to IL‐12/IL‐18 9. Since cNK cells were defective in the liver microenvironment, we tested the role that IL‐10 may play in altering cNK cell function.

Similar to previous studies, we demonstrate that IL‐10 levels can alter NKG2A expression on NK cells 8, 9. We demonstrate that loss of IL‐10 results in lower NKG2A expression on liver trNK, but not splenic or cNK, cells. This correlates with decreased IFN‐γ production upon stimulation through an activating receptor by trNK cells from IL10ko mice as compared to WT mice. This also supports the idea that NKG2A engagement serves as an “educating” receptor that increases IFN‐γ production rather than inhibiting it.

Circulating NK cells from IL10R2 ko (knockout) mice (which do not express the IL‐10 receptor on their cells surface due to deletion of one of the subunits) displayed higher levels of IFN‐γ production as compared to WT mice upon engagement of activating receptors. However, cNK cells from IL‐10 ko mice did not produce more IFN‐γ as compared to WT mice upon engagement of activating receptors. Of note, the IL10R2 receptor subunit makes up a part of several receptors including those for IL‐22, IL‐26, IL‐28, and IL‐29 11, 12, 13. Our study with IL‐10ko versus IL‐10R2 ko mice suggests that IL‐10 may not directly play a role in maintaining the hyporesponsiveness of cNK cells that are transiting through the liver; however, it is possible that one or more of these other cytokines that uses the IL10R2 subunit may play a role in the induction of hyporesponsiveness of liver cNK cells. We did, however, see increased function of trNK cells of the liver in IL10ko mice as compared to WT mice. This suggests that IL‐10 may alter the function of trNK cells, but not cNK cells.

Several key differences could explain the discrepancies between our findings and those of prior studies. In our study, we assess IFN‐γ production and degranulation following stimulation with plate bound antibody to activating receptors rather than cytokine stimulation. In addition, we differentiated the NK cells into splenic, liver trNK and liver cNK, based on CD49a or CD49b expression, when assessing function. Finally, the mechanism in which the NK cells were isolated (perfusion vs. mechanical disruption) from the liver likely plays a role in the ratio of trNK to cNK cells obtained, which could alter how the bulk NK cell population appears to function.

It is now clear that NK cells have the ability to adapt to their environment and show plasticity in their response 5, 50. The liver microenvironment is no exception, as there appear to be a number of mechanisms involved in altering the liver trNK and liver cNK cell responses to activating receptor engagement. These mechanisms are likely in place to prevent unwanted immune responses to the number of harmless antigens that are presented from the gut. These same protective mechanisms, however, may be detrimental to the NK cell's ability to control pathogens in the liver including hepatotropic viruses and tumors (both primary and metastatic). Further understanding of how NK cell tolerance is maintained within the hepatic microenvironment and development of mechanisms to overcome this tolerance in specific circumstances may lead to novel therapeutic strategies, using NK cells, to treat liver pathogens and tumors.

## Materials and Methods

### Mice

The m157‐Tg mouse has been previously described 22. The IL‐10R2 ko mice (B6.129S2‐*Il10rb^tm1Agt^*/J) were obtained from Paul Allen and Tony French (Washington University, St. Louis, MO). The IL‐10ko mice (B6.129P2‐Il10tm1Cgn/J) were purchased from the Jackson Laboratory. Mice were maintained under specific pathogen‐free conditions and used after they reached 8 weeks of age. All animals received humane care according to the criteria outlined in the “Guide for the Care and Use of Laboratory Animals” prepared by the National Academy of Sciences and published by the National Institutes of Health (NIH publication 86‐23 revised 1985). The Animal Studies Committee at Washington University approved all animal studies.

### Antibodies

The following antibodies were obtained from EBioscience (San Diego, CA, USA) or Biolegend (San Diego, CA, USA): APC or PerCPcy5.5‐PK136 (anti‐NK1.1), APC‐Cy7 or PerCP‐Cy5.5‐145‐2C11 (anti‐CD3), Alexa488‐XMG1.2 (anti‐IFN‐γ), APC or PE‐HMα1 (anti‐CD49a), Pacific Blue‐DX5 (anti CD49b), PE‐CD159a (anti‐NKG2A_B6), and streptavidin‐PE. The FITC‐16A11 (anti‐NKG2A) was obtained from Santa Cruz (Dallas, TX, USA). The 3D10 (anti‐Ly49H) and 6H121 (anti‐m157) mAbs were purified from hybridomas by the Protein Production and Purification Core Facility of the Rheumatic Diseases Core Center at Washington University and conjugated to biotin using EZ‐Link Sulfo‐NHS‐LC‐LC‐Biotin (Pierce, Rockford, IL, USA) according to manufacturer's protocol. Purified PK136 (anti‐NK1.1) was purchased from BioXcell (West Lebanon, NH, USA).

### Isolation of hepatic lymphocytes

Livers were removed from mice and passed through a 100 μm filter. The slurry was then passed through a 70 μm filter and transferred into a 15 mL conical tube to be spun at 500×*g* for 5 min. The supernatant was decanted and the pellet resuspended in 8 mL of R2 (RPMI 1640, 2% FCS, 1% Pen/Strep) or R10 (RPMI 1640, 10% FCS, Pen/Strep, l‐glutamine, β‐mercaptoethanol) solution. The 8 mL of liver slurry was mixed with 5 mL of room temperature Percoll (SIGMA, Saint Louis, MO, USA). Following adequate mixing, the solution was centrifuged at 835×*g* for 20 min at room temperature. The supernatant was removed and the remaining pellet was suspended in 10 mL of RBC lysis buffer for 5 min. Lysis was terminated by the addition of 5 mL of R2 or R10 solution, and the tube was centrifuged for 5 min. This pellet was washed with 10 mL of R2 or R10 solution and centrifuged again for 5 min. The resulting pellet of hepatic lymphocytes was resuspended in 1 mL of R10 solution.

### IFN‐γ assays

Splenic cells suspensions were generated as previously described 21, 51 except they were also spun on Percoll gradients and suspended in 10 mL of RBC lysis buffer as described for the isolation of hepatic lymphocytes. Either PK136 mAb (anti‐NK1.1) or 4E4 (anti‐Ly49D) was diluted to 2–4 μg/mL in PBS and placed in 96‐well or 24‐well tissue culture plates (Techno Plastic Product, Saint Louis, MO, USA) and incubated at 37°C for at least 90 min. After incubation, the plates were washed with PBS three times prior to use for stimulation assays. For stimulation of NK cells, splenocytes (1–2 × 10^7^ cells/mL in R10) or hepatic lymphocytes (approximately 1 × 10^7^/mL in R10 for 96‐well plates and approximately 3 × 10^6^/mL in R10 for 24‐well plates) were incubated in wells coated with anti‐NK1.1 or anti‐Ly49D mAb for 1 h and then further incubated in the presence of a 1000‐fold dilution of stock brefeldin A (GolgiPlug, BD Pharmingen, San Diego, CA, USA) for an additional 6–8 h. We used 40 μL of cells per well in the 96‐well plates and 333–500 μL of cells per well in the 24‐well plates. Cells were harvested and stained for surface markers, including NK1.1, CD3, CD49a, NKG2A, CD49b, and Ly49H. Cells were then fixed and permeabilized using Cytofix/Cytoperm solution (BD Pharmingen, San Diego, USA), stained for IFN‐γ and analyzed on the FACSCalibur or FACSCanto (BD Biosciences, San Jose, CA, USA).

### CD107 assays

NK cells were isolated from the liver and spleen and run through a Percoll gradient as described for the IFN‐γ assay. Liver or splenic NK cells were centrifuged onto 24‐well tissue culture plates that had been coated with 6 μg of PK136 for 2 h at 37°C. The NK cells were first stimulated for 1 h at 37°C in the presence of 5 μg/mL αCD107a‐FITC (BD Biosciences). Monensin (Biolegend) was added to achieve 2 μM, and the incubation continued for 5 additional hours. After the stimulation, cells were harvested and stained for surface markers, including NK1.1, CD3, CD49a, NKG2A, and CD49b. Cells were then analyzed by flow cytometry (FCM) on the FACSCanto flow cytometer.

### Statistical analysis

The data were analyzed with Microsoft Excel (Microsoft, Redmond, WA, USA). Unpaired, two‐tailed *t*‐tests or paired *t*‐tests were used to determine statistically significant differences between experimental groups. Error bars in the figures represent the standard error of the mean (SEM).

## Conflict of Interest

None declared.

## Supporting information

Additional supporting information may be found in the online version of this article at the publisher's web‐site

**Figure S1**. Tissue resident (tr) NK cells from WT mice express higher levels of NKG2A than trNK cells from IL10ko mice. The percentage of NK cells expressing NKG2A from WT (black column, n=8) or IL10ko mice (white column, n=4). NKG2A expression was assessed on splenic NK, liver cNK (CD49a+) and liver trNK (CD49a+) cells.

**Figure S2**. Liver trNK cells, but not cNK cells, differ from splenic NK cells in their expression of some inhibitory receptors. Unstimulated murine hepatic and splenic NK cells were stained for Ly49A, Ly49C, Ly49D, Ly49G2, Ly49H, Ly49I and NKG2A and evaluated by flow cytometry.

**Figure S3**. Stimulation with plate bound PK136 does not result in altered CD49a expression on liver NK cells.
