# Supplementary figures and images for: Expression of the inhibitory receptor NKG2A correlates with increased liver and splenic NK cell response to activating receptor engagement
Source: Immun Inflamm Dis. 2017 Mar 24;5(2):177–89. doi: 10.1002/iid3.156 (PMC5418142; doi:10.1002/iid3.156)

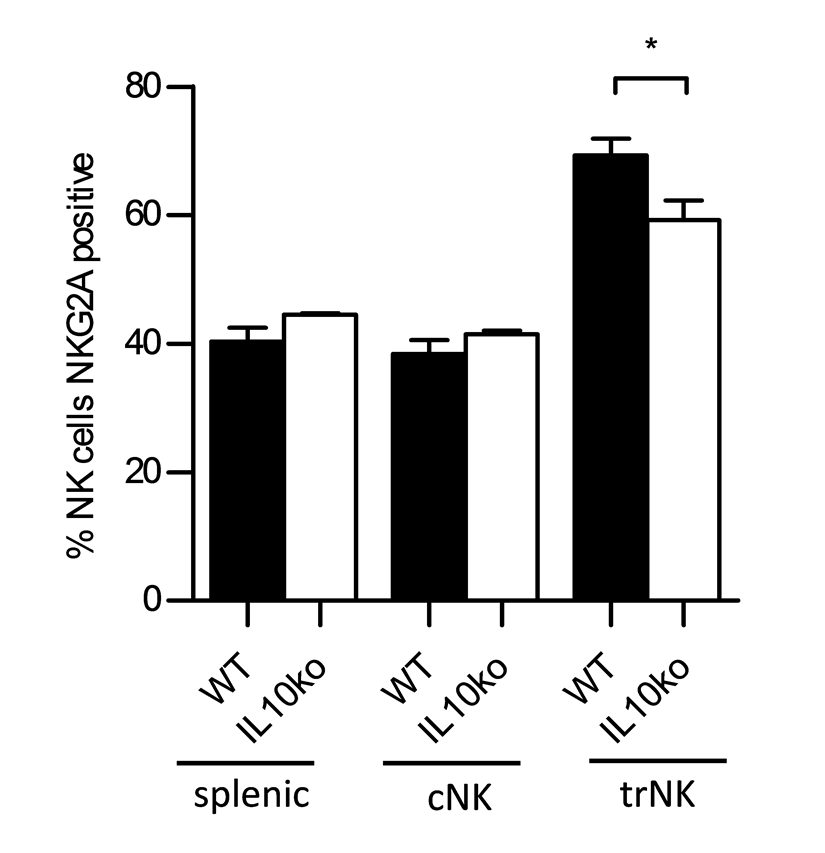

Supplement: Supplementary file 1 — Figure S1. Tissue resident (tr) NK cells from WT mice express higher levels of NKG2A than trNK cells from IL10ko mice. The percentage of NK cells expressing NKG2A from WT (black column, n=8) or IL10ko mice (white column, n=4). NKG2A expression was assessed on splenic NK, liver cNK (CD49a+) and liver trNK (CD49a+) cells. [file IID3-5-177-s001.tif]

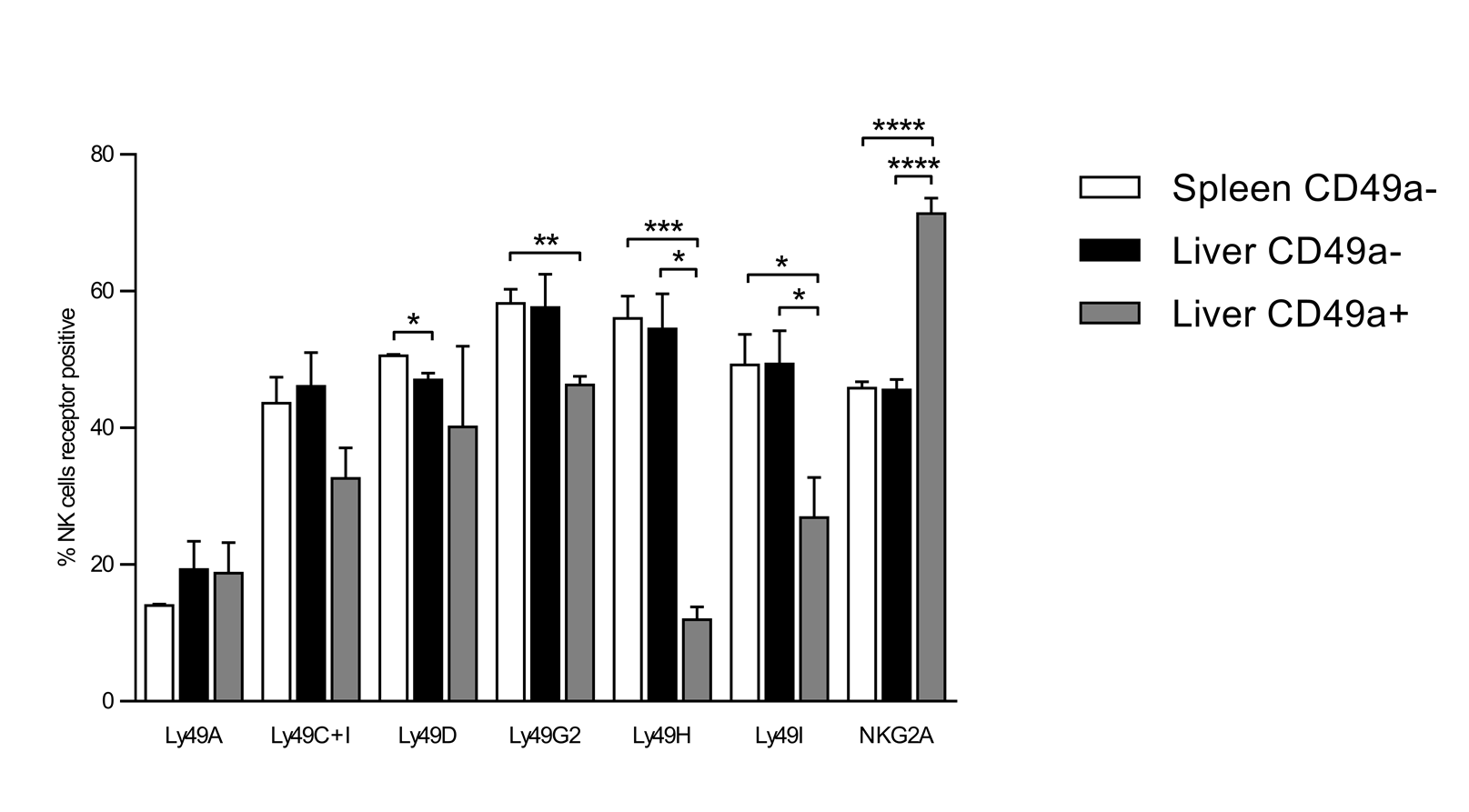

Supplement: Supplementary file 2 — Figure S2. Liver trNK cells, but not cNK cells, differ from splenic NK cells in their expression of some inhibitory receptors. Unstimulated murine hepatic and splenic NK cells were stained for Ly49A, Ly49C, Ly49D, Ly49G2, Ly49H, Ly49I and NKG2A and evaluated by flow cytometry. [file IID3-5-177-s002.tif]

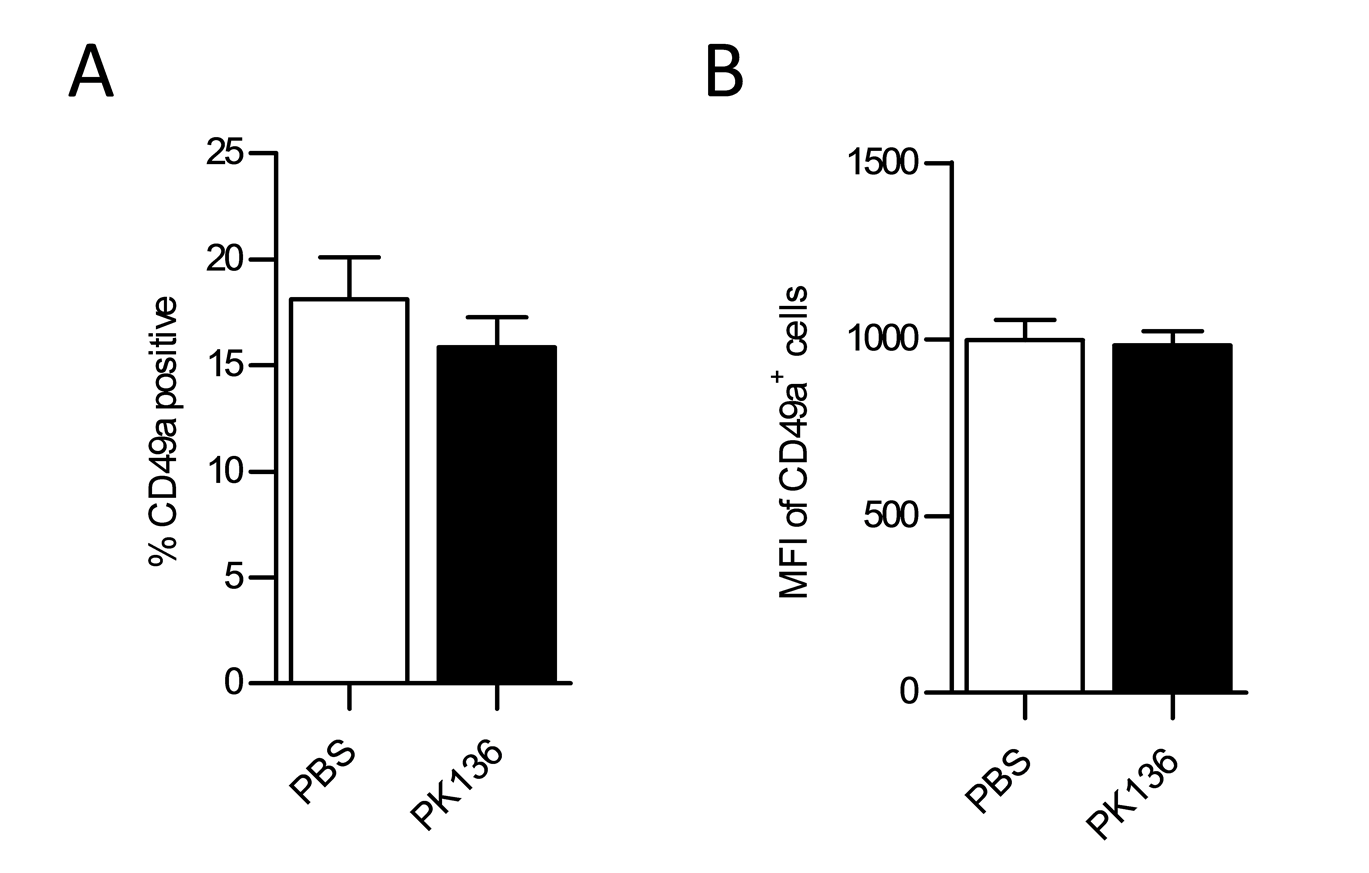

Supplement: Supplementary file 3 — Figure S3. Stimulation with plate bound PK136 does not result in altered CD49a expression on liver NK cells. [file IID3-5-177-s003.tif]
